# Sensitivity assessment of workflows detecting rare circulating cell-free DNA targets: A study design proposal

**DOI:** 10.1371/journal.pone.0253401

**Published:** 2021-07-06

**Authors:** Thorsten Voss, Andrea Ullius, Maike Schönborn, Uwe Oelmüller

**Affiliations:** R&D Department, QIAGEN GmbH, Hilden, Germany; University of Helsinki: Helsingin Yliopisto, FINLAND

## Abstract

The field of liquid biopsy has seen extensive growth in recent decades, making it one of the most promising areas in molecular diagnostics. Circulating cell-free DNA (ccfDNA) especially is used as an analyte in a growing number of diagnostic assays. These assays require specified preanalytical workflows delivering ccfDNA in qualities and quantities that facilitate correct and reliable results. As each step and component used in the preanalytical process has the potential to influence the assay sensitivity and other performance characteristics, it is key to find an unbiased experimental setup to test these factors in diagnostic or research laboratories. We defined one such setup by using blood from healthy subjects and commercially available products for blood collection, spike-in material, ccfDNA isolation, and qPCR assays. As the primary read-out, we calculated the probit model-based LOD95 (limit of detection of the 95^th^ percentile) from the qPCR assay results. In a proof of principle study we tested two different but widely used blood ccfDNA profile stabilization technologies in blood collection tubes, the Cell-Free DNA BCT and the PAXgene Blood ccfDNA Tube. We tested assays for three different *EGFR* gene mutations and one *BRAF* gene mutation. The study design revealed differences in performance between the two tested technologies for all four mutations. In conclusion, we successfully established a blueprint for a test procedure capable of verifying and validating a liquid biopsy workflow from blood collection to the analytical result.

## Introduction

The application of cell-free nucleic acids derived from body fluids in diagnostic procedures is one of the most promising areas of innovation in molecular diagnostics in recent decades. DNA from blood plasma, which is defined as circulating cell-free DNA (ccfDNA), is used in an increasing number of disease areas and related fields, because of the many advantages coupled to this analyte and its exploitation (for reviews see references [1–4]). As ccfDNA is a derivate from the blood circulation, genetic information of all body areas can be retrieved in a frequent sequence of minimally invasive, standardized blood collections. At the moment, liquid biopsy tests applied for cancer diagnostics are primarily used as complementary methods to conventional biopsies. Nevertheless they are gaining importance as a true alternative to tissue biopsies, which are limited to the region of excision as well as in the frequency of repetitions [5–8] and for post-surgery patient surveillance [9].

Even though the knowledge about the existence of ccfDNA reaches back to the first half of the 20^th^ century [10], its use as a diagnostic tool was first established between the late 1990s and the first decade of the 21^st^ century in the field of non-invasive prenatal testing (NIPT) [11,12]. Most NIPT applications focus on the detection of chromosomal abnormalities by counting the number of chromosomal fragments in the ccfDNA and detecting abnormal ratios as diagnostic evidence. Therefore, it is key that these ratios are not artificially disturbed and can be reliably detected. As the total amount of ccfDNA in the maternal plasma is very low in most cases (<10 ng per ml of plasma) and the DNA derived from the fetus is only a small fraction of this quantity, the post collection release of additional genomic DNA (gDNA) from maternal nucleated blood cells could overlay or dilute the original ccfDNA. This can lead to unreliable usage for NIPT testing, particularly in stored specimen [13,14]. This requires implementing measures to avoid post collection gDNA release during preanalytical workflow steps before performing the diagnostic assay [15,16]. Such pre-analytical workflows comprise all steps from blood collection, storage, transport and processing and ends with the ready to use isolated ccfDNA.

In addition to NIPT, the use of ccfDNA as a basis for diagnosis, disease monitoring, and therapy decisions has gained increased importance in the field of oncology (for review see references [17–20]). Here the extremely low amounts of target molecules are often the limiting factor, for example in early cancer stages or during minimal residual disease monitoring [21]. The low abundance in combination with gDNA dilution from lysing white blood cells makes every target count. Any bias introduced by the preanalytical steps could produce misleading results [22–24]. In other words, the sensitivity of a ccfDNA diagnostic assay is largely determined by the initial specimen integrity and processing, and the requirements of the assay determine the needs and measures in the preanalytical workflow [25–27].

Because of the indisputable importance of the preanalytical steps for all areas where molecular diagnostic procedures are used [28], the European Union funded two large consortia projects called SPIDIA (standardisation and improvement of pre-analytical procedures for in-vitro diagnostics, 2008–2013) and SPIDIA4P (2017–2021), [29,30] to develop dedicated, optimized, and standardized methods and devices as well as operating procedures. Outputs of these consortia include CEN Technical Specifications which are subsequently progressed to ISO International Standards [31] such as the recently released Blood ccfDNA standard *ISO 20186–3 Molecular in vitro diagnostic examinations–Specifications for pre-examination processes for venous whole blood–Part 3*: *Isolated circulating cell free DNA from plasma*:*2019*, developed via the ISO Technical Committee “Clinical laboratory testing and in vitro diagnostic test systems”. Moreover, these consortia enabled the integration and optimization of existing and novel technologies, methods, standards and products to standardized diagnostic workflows.

Compliance to this ISO standard is the basis of sound and reliable ccfDNA-based diagnostic procedures, but standards give only the framework and still allow diagnostic laboratories to use different technologies in both, the preanalytical and analytical parts of their workflows. Therefore, test designs are necessary to form an unbiased opinion of different technologies and to facilitate a data-based decision which satisfies individual laboratory’s specific diagnostic needs. These tests need to have the ability to discriminate between single components and working steps in the planned workflow, as each of these criteria could have a significant influence on the diagnostic result [32,33].

Looking back to the early years of this century, the development of highly sensitive diagnostic assays and workflows for the detection of viruses like HIV, HBV and HCV were needed to accompany therapies. To judge the product’s sensitivity, the limit of detection (LOD) of quantitative PCR (qPCR) assays were identified as the parameter of choice when supported by statistical methods based on probit models [34–36]. As the status of ccfDNA-based diagnostics in the oncology field is currently at a comparable developmental stage, we decided to design and test a study setup that could help guide decision making in the construction of ccfDNA diagnostic workflows, especially in the oncology field.

In such a workflow, one key element is the choice of dedicated blood collection tubes as described and recommended by the ISO Standard 20186–3. Standard tubes for plasma analyses using anticoagulants such as EDTA have a very limited potential to prevent apoptosis and other cell lytic processes. Small quantities of genomic DNA begin to enter the blood immediately after collection and increase further during storage and transport, thus diluting dedicated ccfDNA molecules targeted by analytical tests. Cooling of EDTA blood samples may reduce this post collection gDNA release for short periods, e.g. between four to six hours [37,38] or less depending on the analytical test requirements. Heparin, another anticoagulant, has an even worse effect on prevention of gDNA release, and serum samples contain significant amounts of gDNA immediately after collection in nearly all cases. This subsequently makes the use of such samples for ccfDNA analysis problematic [39]. Because of this clear need for blood stabilization to avoid post collection release of gDNA, unique additive chemistries for blood collection tubes were developed to preserve white blood cells and help prevent the release of gDNA (reviewed by [23,32]).

The aim of this research study is to set up a generic test procedure capable of verifying and validating single components of a liquid biopsy workflow from blood collection to the analytical result, which can be used as a blueprint for other researchers and clinicians to evaluate their workflows. We designed a test scenario which mimics the daily routine in a standard diagnostic laboratory using the LOD95 (LOD of the 95^th^ percentile) of two standard qPCR assays. The analysis targeted three common mutations of the *EGFR* gene and one mutation of the *BRAF* gene as readouts [40]. We implemented this new LOD95 test design by using two widely used but principally different blood ccfDNA profile stabilizing technologies; the Cell-Free DNA BCT (blood collection tube; Streck Inc.) and the PAXgene Blood ccfDNA Tube (PreAnalytiX). Designated vendor recommended conditions for both tube technologies were used to minimize the bias caused by the experimental conditions.

## Material and methods

The study was carried out according to the *ISO Standard 20186 Molecular in vitro diagnostic examinations–Specifications for pre-examination processes for venous whole blood–Part 3*: *Isolated circulating cell free DNA from plasma*:*2019*.

### Study design

**Fig 1:**
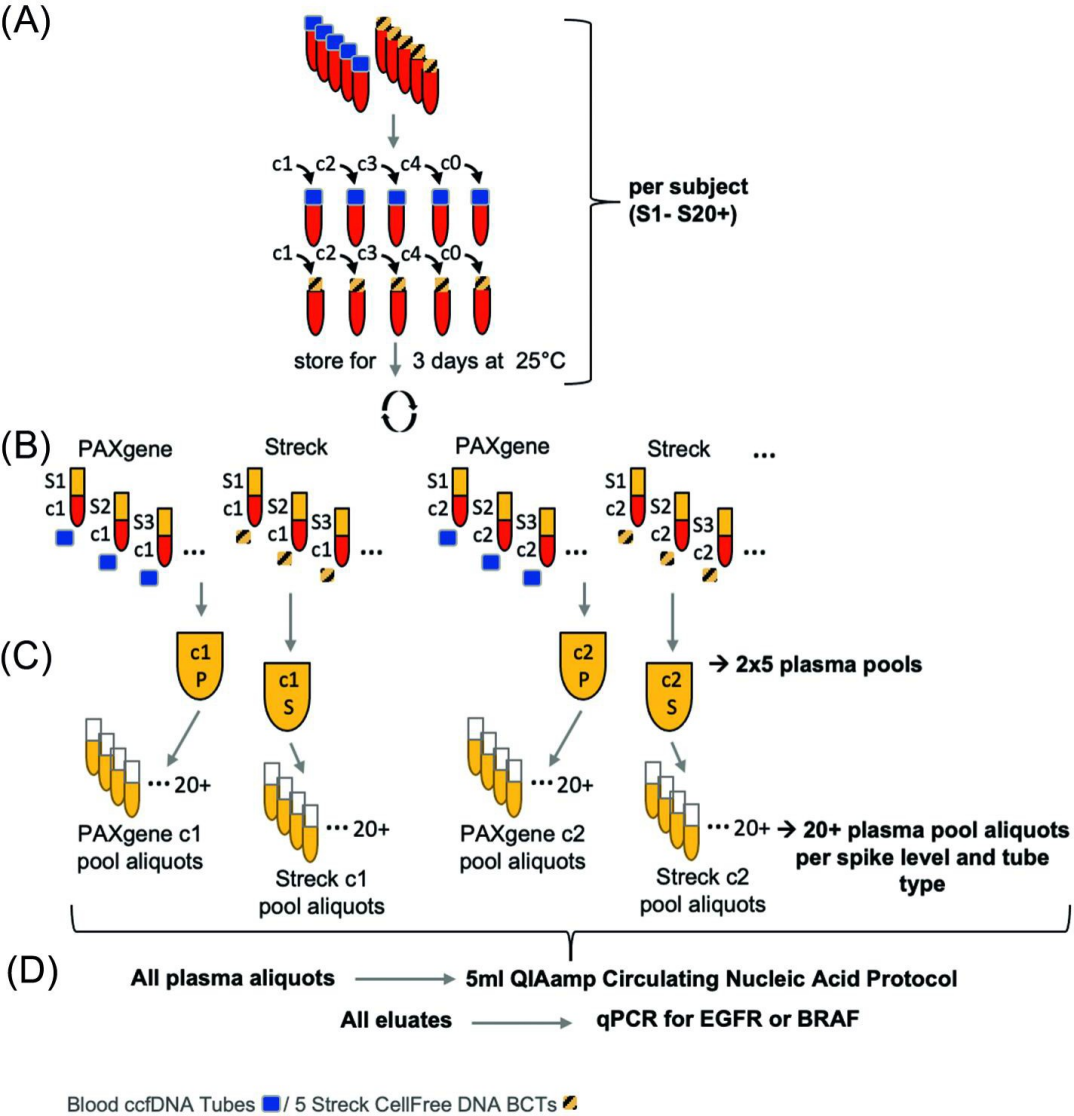
Flow chart of the experimental setup. (A) Blood collection: Blood from at least 20 subjects per experiment (S1-S20+) was drawn in random order with 10 replicate tubes per subject divided in 5 PAXgene Blood ccfDNA Tubes and 5 Streck CellFree DNA BCTs. Only completely drawn tubes (10ml blood) were included. (B) Spike-in & storage: 4 concentrations (c1-c4) of spike-in material was pipetted in the blood directly after draw, The fifth tube was left un-spiked as negative control (c0). All tubes were stored for 3 days at 25°C. (C) Plasma-generation & -pooling: Plasma was separated with a centrifugation protocol recommended by the tube suppliers. Complete plasma was removed from each tube. All plasma aliquots of one spike-level within one tube type were pooled. Plasma volume was measured of each pool and calculation of tube equivalent aliquot volume was done. (D) ccfDNA isolation, PCR assays and LOD calculation: Plasma pools were split in 20+ aliquots with a tube equivalent volume each. CcfDNA was isolated from each aliquot using the QIAamp Circulating Nucleic Acid Kit and the 5ml plasma input protocol. For eluate QC all eluates were analyzed by Qubit and Bioanalyzer measurements. QPCR assays targeting the spiked *EGFR* or *BRAF* gene mutations were run with all eluates. Probit based calculation of the LOD95 values were carried out for all qPCR (sub-)assays.

The objective of the study was to evaluate a study design for the verification and validation of single compounds of preanalytical ccfDNA-based workflows and analytical sensitivity of downstream assays in a setting that mimics clinical laboratory routine. To avoid misleading effects like donor-to-donor variation, extreme blood storage conditions, sub-optimal centrifugation protocols, and different ccfDNA isolation procedures which can interfere with the influence of the single component tested on the readout, the setup was fixed in all these aspects and variations were reduced to an absolute minimum. The study design is shown in Fig 1.

Briefly, blood was collected in both tube types from at least 20 donors per independent experiment. The blood draw was randomized to avoid effects caused by tube position, timing, and rate of fill. All tubes were visually inspected for correct blood volume, and all donors with single underfilled tubes were excluded from the experiment. This exclusion is the primary reason for the varying number of donors per experiment. Within two hours after draw, four tubes per donor and tube type were spiked with four different amounts of ccfDNA-sized DNA standards. These fragments carried various common mutations of the *EGFR* and *BRAF* genes and had sizes of around 170 bp to mimic the main portion of naturally occurring ccfDNA, corresponding to a mononucleosomal DNA fragment. The fifth tube per donor and tube type remained un-spiked. All tubes were incubated for three days at room temperature to simulate a transport or over-weekend situation. Plasma was generated via centrifugation and as much plasma as possible was harvested from all tubes following incubation, without disturbing the buffy coat. All plasma aliquots with the same spike-in level and tube type were pooled, and the individual volumes of these pools were measured. The pool with the lowest volume was identified and divided by the number of subjects. The resulting value specified the plasma volume for every aliquot within one tube type and spike level which is referred to as “tube equivalent aliquot volume” (V_A_, Eq 1):

$$V_{A}[\mathrm{ml}]=\frac{\mathrm{volume}\;\mathrm{pool}\;[\mathrm{ml}]}{\mathrm{number}\;\mathrm{of}\;\mathrm{subjects}}$$ (1)

All plasma pools were aliquoted with the V_A_ and the resulting tube equivalent aliquots were stored frozen at -20°C until use.

As recommended by the suppliers, frozen PAXgene plasma aliquots were thawed at 30°C, while Streck plasma was thawed at room temperature. For ccfDNA isolation, the identical kit and protocol was used, based on the ability to process the different plasma aliquot volumes generated from the pools. The quantity and quality of ccfDNA was assessed for all resulting eluates. The recovery of the spiked mutations was subsequently analyzed by two qPCR systems. The LOD95 for all four qPCR assays − targeting three mutations in the *EGFR* gene and one mutation in the *BRAF* gene − was determined as the final assessment criterion for whole workflow sensitivity.

### Blood samples and DNA standards

Ethics approval was granted from ethics committee of North Rhine Westphalia, Germany (2007389). In accordance with this ethics approval, blood was collected via the internal QIAGEN blood collection service in Hilden, Germany between August 2019 and August 2020 under the supervision of the company’s medical officer. All subjects were volunteers and employees of QIAGEN’s site in Hilden, Germany and by that under constant medical review as requested by German employment law. The blood collection service accepted subjects aged between 18 and 68 years who underwent an additional, blood donation specific medical examination upfront their first donation. For all subjects the hematocrit value was monitored monthly and need to be equal or above 12,5 g/dL for women and 13,5 g/dL for men. Exclusion criteria for subjects were a body weight below 50 kg, a body mass index equal or below 18, and a known pregnancy. Prior to every blood donation the subjects gave their written consent. They had to be apparently healthy at the time of collection. According to the ethics approval all blood samples where anonymized before they were handed over to the laboratory researchers. Overall a group of 174 subjects were enclosed in this study.

For each experiment, whole blood was collected and five replicate samples per subject were drawn into Cell-Free DNA BCTs CE (Streck, Omaha, NE) and five replicate samples per subject into PAXgene Blood ccfDNA Tubes CE-IVD (PreAnalytiX, Hombrechtikon, Switzerland). All blood draws were carried out according to the manufactures’ instructions for use. *EGFR*-Multiplex 5% AF cfDNA Standard carrying the mutations T790M, L858R and the deletion in exon 19 (Del Ex19) or the 5-Gene-Multiplex 5% AF cfDNA Standard carrying the *BRAF* mutation V600E (both SensID, Rostock, Germany) were used to spike the samples. Both standards consist of DNA fragments in the size of mononucleosomal fragments between 151 bp and 181 bp with the main peak at 167 bp in length. After being spiked, the tubes were recapped and carefully inverted 10 times.

For formaldehyde testing, blood was drawn into a 10 ml K_2_EDTA tube (BD, Franklin Lakes, NJ).

### Plasma generation and pooling

Following the manufacturer’s recommendations, PAXgene Blood ccfDNA Tubes were centrifuged first at 1,900 x g and room temperature for 15 minutes. After complete plasma transfer into a secondary tube, a second round of centrifugation was carried out at 1,900 x g and room temperature for 10 minutes, followed by another transfer of the plasma into a fresh tube. Henceforth, these plasma samples are called PAXgene plasma samples.

For Cell-Free DNA BCTs, a first centrifugation was done at 1,600 x g and room temperature for 10 minutes as written in the tube product circular (protocol 2). After complete plasma transfer into a secondary tube, a second round of centrifugation was carried out at 16,000 x g and room temperature for 10 minutes, followed by another transfer of the plasma into a fresh tube. As with the PAXgene plasma samples, these samples are named Streck plasma samples.

EDTA plasma was generated from blood collected in K_2_EDTA tubes directly after draw by a first centrifugation at 1,900 x g and 4°C for 10 minutes and a second centrifugation for 10 minutes at 16,000 x g and 4°C.

### Plasma DNA preparation method

To extract ccfDNA from all plasma aliquots, the QIAamp Circulating Nucleic Acid Kit (QIAGEN, Hilden, Germany) with the protocol “Purification of Circulating Nucleic Acids from 4 ml or 5 ml Serum or Plasma” was used. While PreAnalytiX explicitly claims compatibility with this kit in the PAXgene Blood ccfDNA Tube instructions for use, Streck is stating that extraction of cell-free plasma DNA can be accomplished using most commercially available kits in the Cell-Free DNA BCT instructions for use. In the majority of papers listed as references for the Cell-Free DNA BCT on the Streck homepage [41] the QIAamp Circulating Nucleic Acid Kit was successfully used. With therefore we felt comfortable to use the QIAamp Circulating Nucleic Acid Kit for isolating ccfDNA from plasma generated from both tubes types. For Streck plasma samples, the incubation time with proteinase K was extended to one hour as recommended by the tube provider. If the plasma aliquot volume was less than 5 ml, the remaining volume was filled with PBS (ThermoFisher Scientific, Waltham, MA). An elution volume of 60 μl buffer AVE was used.

### Formaldehyde testing

Formaldehyde content in plasma was analyzed and quantified using Quantofix Formaldehyde strips (Macherey-Nagel, Düren, Germany) with a serial dilution of 10% neutral buffered formalin solution (Sigma Aldrich Inc., St. Louis, MO) as reference.

### Analyzing methods

The ccfDNA yield was quantified using a Qubit 2.0 Fluorometer and Qubit 1x dsDNA HS Assay-Kit (Thermo Fisher Scientific, Waltham, MA). Sample quality was analyzed by capillary electrophoresis using a 2100 Bioanalyzer instrument and the associated High Sensitivity DNA Kit (Agilent, Santa Clara, CA).

The recovery of spiked-in material was analyzed with qPCR.

*EGFR* mutations T790M, Del Ex19, and L858R spiked in the blood using *EGFR*-Multiplex 5% AF ccfDNA standard were analyzed via qPCR by using the *therascreen EGFR* Plasma RGQ PCR Kit (QIAGEN, Hilden, Germany). Spiked 5-Gene-Multiplex 5% AF cfDNA Standard, carrying *BRAF* V600E mutant, were detected with the *therascreen BRAF* RGQ PCR Kit (QIAGEN, Hilden, Germany). Both assays were run on a Rotor-Gene Q instrument (QIAGEN, Hilden, Germany).

For LOD95 calculation the probit-model-based software Arcus Quickstat Statistical Software Version 1.1.0.137 (Arcus Biomedical) was used.

## Results

Overall, five studies were carried out using *EGFR* gene and three using *BRAF* gene DNA standards as spike-in material. A total of 174 donors were utilized for the studies—108 subjects for the *EGFR* mutation and 66 subjects for BRAF mutation analysis.

### Generated plasma volumes and ccfDNA yield

Apart from the essential LOD95 qPCR readouts for the two tested preanalytical workflows which are shown below, additional characteristics of the plasma samples were collected during testing. The ccfDNA yield and quality from each tube was of particular interest.

The plasma volume of blood samples stored in PAXgene Blood ccfDNA Tubes was 41% higher (5.5 ml ± 0.16 ml) than the plasma volume obtained from blood stored in Cell-Free DNA BCTs (3.9 ml ± 0.31 ml) based on 870 individual tubes collected per tube type. In our opinion this difference in plasma volume for both tube types is mainly caused by different volumes of stabilization agent pre-filled in the two tube types. While this volume for the PAXgene Blood ccfDNA Tubes is 1.5ml [42] Streck is not disclosing this value for their Cell-Free DNA BCTs. We determined this volume to be in a range between 100 and 150μl by pipet measurement. The resulting delta in volume of the stabilization solutions in the tubes is 1.35 to 1.40 ml. This difference is close to the mean difference in plasma volume we could harvest per tube type which is 1.6 ml. As the blood draw volume for both tubes was identical (10 ml), we decided to calculate individual V_A_ for the different pools to have a fair comparison. Consequently, we compared both workflows at the level of blood rather than plasma volume.

**Fig 2:**
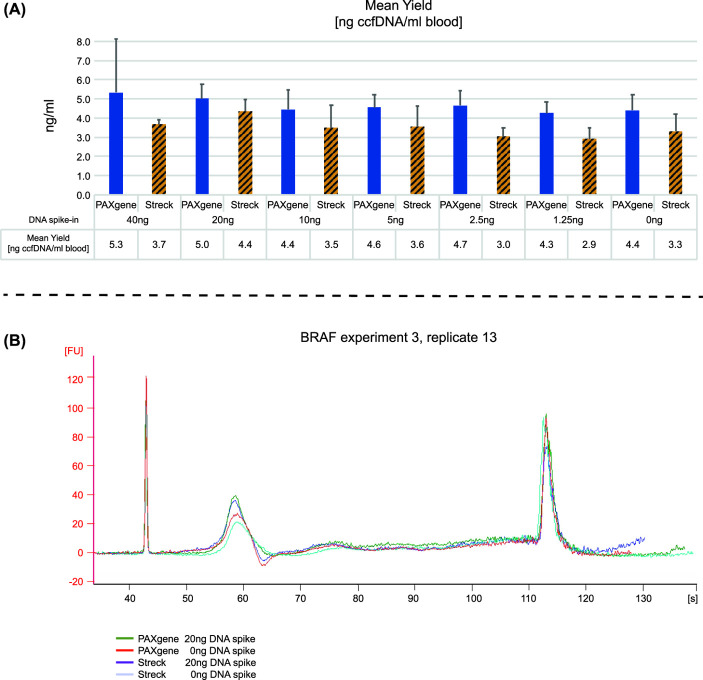
Quality control: CcfDNA yield and fragment distribution. (A) Qubit 1x dsDNA HS Assay-Kit. (B) Exemplary Bioanalyzer electropherograms. A 2100 Bioanalyzer instrument and the associated High Sensitivity DNA Kit were used. FU stands for fluorescence units and s for seconds run time.

Comparable yields were reported during ccfDNA quantification of extracted PAXgene and Streck plasma samples. Mean values of the un-spiked controls of all eight experiments were calculated. With n = 174 the mean yield was 4.4 ± 0.8 ng ccfDNA per ml blood for PAXgene plasma samples and 3.3 ± 0.9 ng ccfDNA per ml blood for Streck plasma samples (Fig 2A). An effect of additional amount of the spiked-in material on the overall ccfDNA yield could not clearly be distinguished compared to the un-spiked pools. Details on plasma volumes and yields of the single experiments can be found in S1 Fig and the accompanying S1 Table.

Both spiking materials had no visible effect on the fragment size distribution in the ccfDNA eluates, as Bioanalyzer capillary electrophoresis reveals (Fig 2B exemplary electropherograms from spiked and un-spiked samples). In all analyzed samples, the main mononucleosomal ccfDNA peak was clearly visible. The samples showed very low amounts of multimers of these fragments. No additional peaks and only minor amounts of high molecular weight DNA could be detected. This indicates the efficient white blood cell stabilizing capability of the tubes and no interference of the spiking process in their mode of action.

### Analytical sensitivity of *EGFR* gene and *BRAF* gene mutation detection

**Fig 3:**
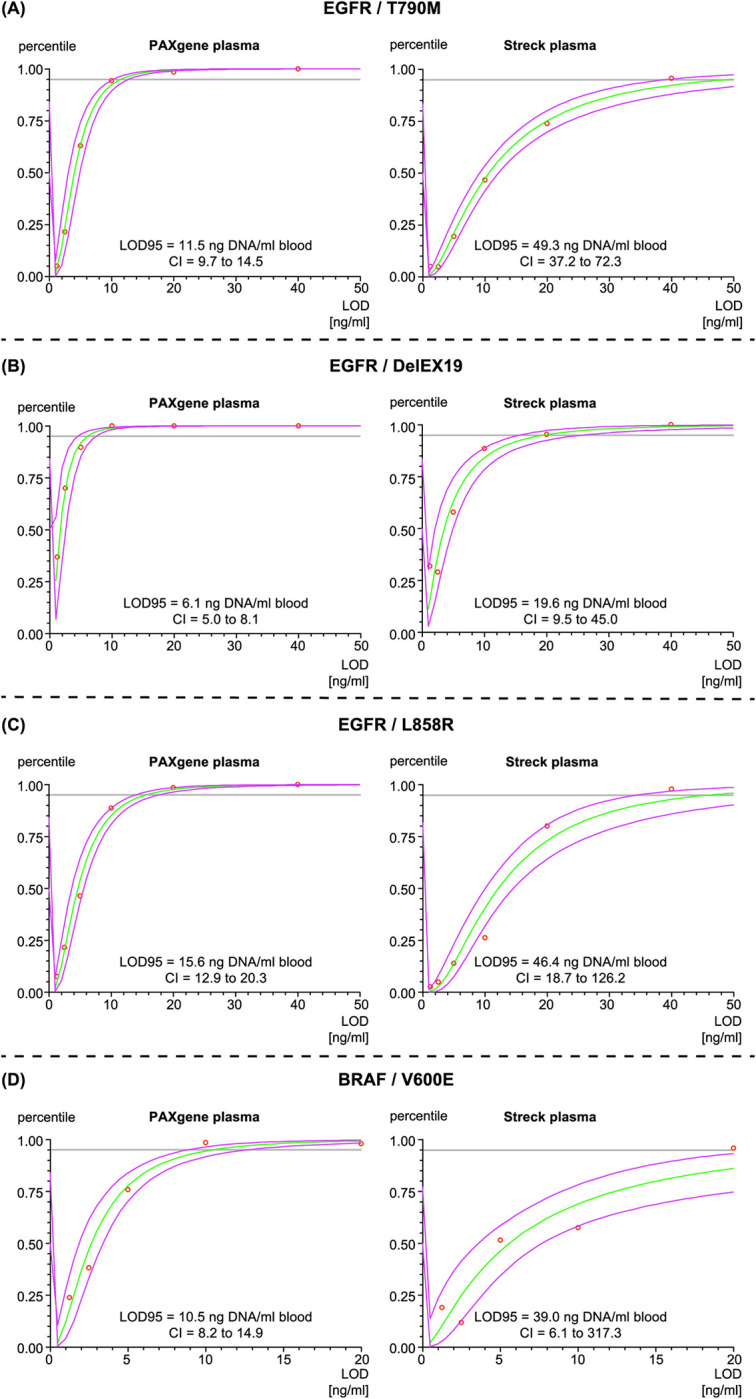
Detection rates and confidence intervals. Calculations and graphs were executed by probit-model-based Arcus Quickstat Statistical Software Version 1.1.0.137. Green lines represent the calculated LOD and purple lines represent the corresponding confidence intervals. CI stands for confidence interval.

Two commercially available qPCR assay systems were used to detect the recovery of the rare target sequences spiked into the blood samples. We defined the LOD95 as the main readout to judge the sensitivity of the two workflows. The detection rates of the three *EGFR* gene mutations spiked with the DNA standard material are shown in Fig 3A–3C. For all three assays, mutations hit rates were lower for samples collected and stabilized in Cell-Free DNA BCTs compared to those originated from PAXgene Blood ccfDNA Tubes. The LOD95 values are expressed in ng DNA spike-in needed per ml blood to achieve a positive assay result for 95% of the samples. For samples collected in PAXgene Blood ccfDNA Tubes, 11.5 ng DNA/ml blood for T790M, 6.1 ng DNA/ml blood for Del Ex19, and 15.6 ng DNA/ml blood for L858R would be needed. For samples stabilized in Cell-Free DNA BCTs, 49.3 ng DNA/ml blood for T790M, 19.6 ng DNA/ml blood for Del Ex19, and 46.4 ng DNA/ml blood for L858R had to be spiked in for appropriate detection. The higher LOD95 values observed for Streck tubes with the *EGFR* gene mutations were reproduced by three additional experiments involving DNA spike-in material carrying the *BRAF* gene mutation V600E. Here we found a LOD95 of 10.5 ng DNA/ml blood (PAXgene) and 39.0 ng DNA/ml blood (Streck) respectively (Fig 3D).

### Formaldehyde detection in plasma

As we found distinct differences in the sensitivity of both workflows linked to the blood collection tubes used for ccfDNA stabilization, we investigated potential subjacent differences in the ccfDNA stabilization mode of action of both tubes. The detailed process of how stabilization additives preserve blood cells is proprietary to the respective companies. With regard to Streck, several patents (US000008586306B2, US000009657227B2, US000010144955B2, US000010294513B2, EP000002814981B1, EP000002228453B1) indicate that blood cell stabilization in the Cell-Free DNA BCTs may be facilitated by formaldehyde releasing substances like imidazolidinyl or diazolidinyl urea.

**Fig 4:**
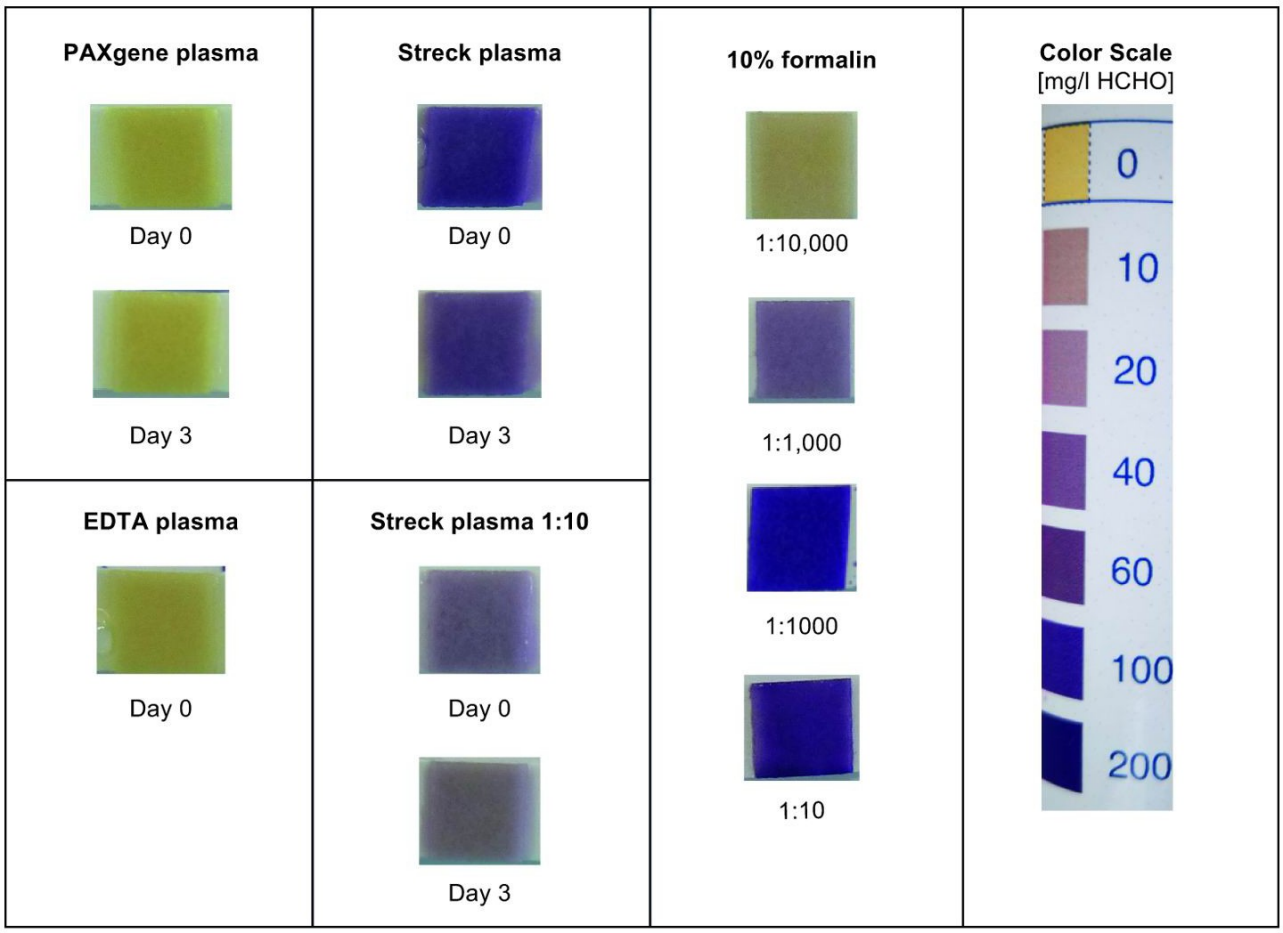
Formaldehyde detection. Formaldehyde content in plasma was analyzed and quantified using Quantofix Formaldehyde stripes.

With therefore we applied a commercially available, semi-quantitative strip test for formaldehyde detection in the plasma prepared from blood collected in the used tube types. Streck plasma clearly displayed a color change similar to that of a control solution with formalin dissolved in water (Fig 4), while no formaldehyde could be detected in PAXgene plasma.

## Discussion

Sensitivity of an analytical assay is strongly dependent on the analyte integrity and quality. This is especially true for assays which are designed to detect very low abundance target molecules, as they are used in ccfDNA based research as well as diagnostics in the oncology field [6,43]. Therefore, the preanalytical workflow which delivers these analytes is the key part for correct and reliable analytical test results. In other words, the test defines the requirements which the preanalytical workflow has to fulfill for enabling the specified test performance. This principle view is also reflected in the ISO International Standard 20186–3. Thus, a thorough and unbiased analysis of the single components and steps in a whole workflow is key for a responsible decision on components to be used and steps to be specified.

In several prior studies, comparisons between single components or steps were published which are part of preanalytical processes used for ccfDNA exploitation. These comparisons included evaluations of sample type [39,44,45], different collection tubes [46–48], centrifugation conditions [49–51], or ccfDNA extraction chemistries [22,33]. The previous studies gave insights into certain aspects of the workflow, but their setups are not aligned with ISO 20186–3, have only a limited sample size, deal with challenging sample material, and/or use a complex and difficult to reproduce experimental setup [32,33].

To our knowledge, the experimental design we used in this study is the first generic setup that can be used to determine differences in preanalytical blood ccfDNA workflows including stabilization and isolation, as well as for adjacent analytical assays. As we used blood from healthy subjects, commercially available components, and the widely accepted LOD95 value as the readout, professional research and diagnostic laboratories should be able to set up experiments to test their planned or existing workflow components by using our methodology as a blueprint.

The aim of this study was to show the proof of principle of the generic evaluation workflow we designed. Therefore we tested one key component, the ccfDNA profile stabilization technology incorporated in a blood collection tube, with our setup. While both technologies/tubes displayed good performance with respect to their main tasks -stabilizing white blood cells and preventing gDNA release, differences in the detection rates of the target sequences between samples derived from these two tube types were clearly demonstrated. This result proves that the impact of differences between components of the workflow can be clearly detected by this setting.

Sensitivity is the key factor for early diagnosis and maximum patient benefit, particularly in cancer screening and minimal residual disease monitoring [52,53]. The differences in the LOD95 between the tested tube types demonstrates that the choice of the ccfDNA stabilization technology can influence the sensitivity of the assay in use.

Beside other ccfDNA stabilization tubes, the Cell-Free DNA BCTs have been used successfully in the NIPT field for many years. The assays used in the prenatal field are dependent on the correct ratio of fragments from the different chromosomes. This is provided by the stabilization function of the Cell-Free DNA BCT technology as well as the PAXgene Blood ccfDNA Tube technology. The amount of target sequences is not as limited as for most assays used in oncology. Therefore, sensitivity is not the most important requirement for NIPT assays.

To explain the result differences observed between the two tube stabilization technologies, we investigated the mode of action of Streck’s and PAXgene’s stabilization chemistry. Das and coworkers [54] failed in detecting free formaldehyde in plasma generated from blood collected in the Cell-Free DNA BCTs using carbon-13 NMR. In contrast to this finding we were able to detect the presence of aldehyde species in Streck plasma with a simple strip test. Stabilization of white blood cells in the Cell-Free DNA BCTs is presumably achieved by these reactive substances. According to patents issued by Streck (US000008586306B2, US000009657227B2, US000010144955B2, US000010294513B2, EP000002814981B1, EP000002228453B1), chemicals like diazolidinyl or imidazolidinyl urea, which release formaldehyde over time, are part of the claimed stabilization solution. Formaldehyde has been known to introduce crosslinks between biomolecules. Crosslinked membranes of white blood cells prevent the disintegration of cells and the release of gDNA [23]. As a side effect, other molecules in the blood sample- including ccfDNA- are crosslinked as well. The DNA-crosslinks can be partially reversed by special treatments during the ccfDNA isolation procedure, for example by a long proteinase K digestion. The remaining crosslinks have the potential to impair the amplification efficiency of ccfDNA molecules or other assay related processes, which are central to most downstream methods such as quantitative or digital PCR and next generation sequencing (NGS) library preparations. In contrast, no aldehyde molecules could be detected in PAXgene tubes, demonstrating a principle difference between the two blood ccfDNA profile stabilization technologies. According to information on the PreAnalytiX homepage the PAXgene Blood ccfDNA technology is formaldehyde-free and non-crosslinking.

In conclusion, we established a blueprint for a test procedure which is capable of validating a liquid biopsy workflow from blood collection to the analytical results for ccfDNA related workflows. A similar approach could be used for other liquid biopsy related analytes like ccfRNA or circulating tumor cells. Furthermore, researchers working on liquid biopsy workflows using other sample materials beside plasma − including urine, saliva or CSF (cerebrospinal fluid) − could use this setup with small adaptations to test their components. In combination with existing and upcoming new preanalytical ISO and CEN standards, a thorough characterization of workflows will enhance and establish the use of liquid biopsies as a routine diagnostic, including applications beyond the oncology field.

## Supporting information

S1 Fig Plasma volumes. (A) Plasma volumes of single experiments. (B) Plasma mean volumes.
(TIF)

S1 Table CcfDNA yields of single experiments. Yields were determined by using a Qubit 2.0 Fluorometer and Qubit™ 1x dsDNA HS Assay-Kit.
(TIF)
