# Supplementary figures and images for: Sensitivity assessment of workflows detecting rare circulating cell-free DNA targets: A study design proposal
Source: PLoS One. 2021 Jul 6;16(7):e0253401. doi: 10.1371/journal.pone.0253401 (PMC8260181; doi:10.1371/journal.pone.0253401)

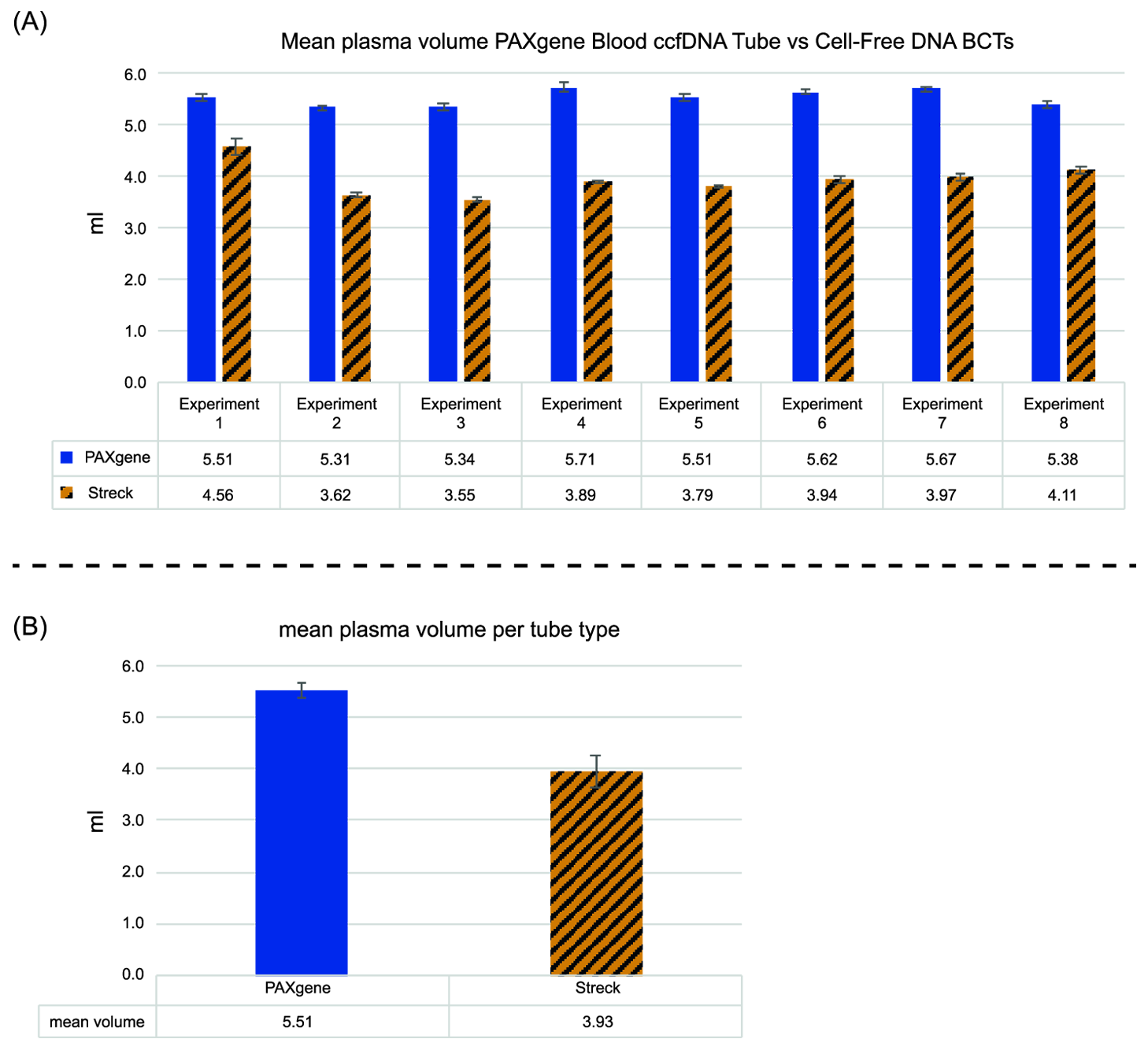

Supplement: S1 Fig — (A) Plasma volumes of single experiments. (B) Plasma mean volumes. (TIF) [file pone.0253401.s001.tif]

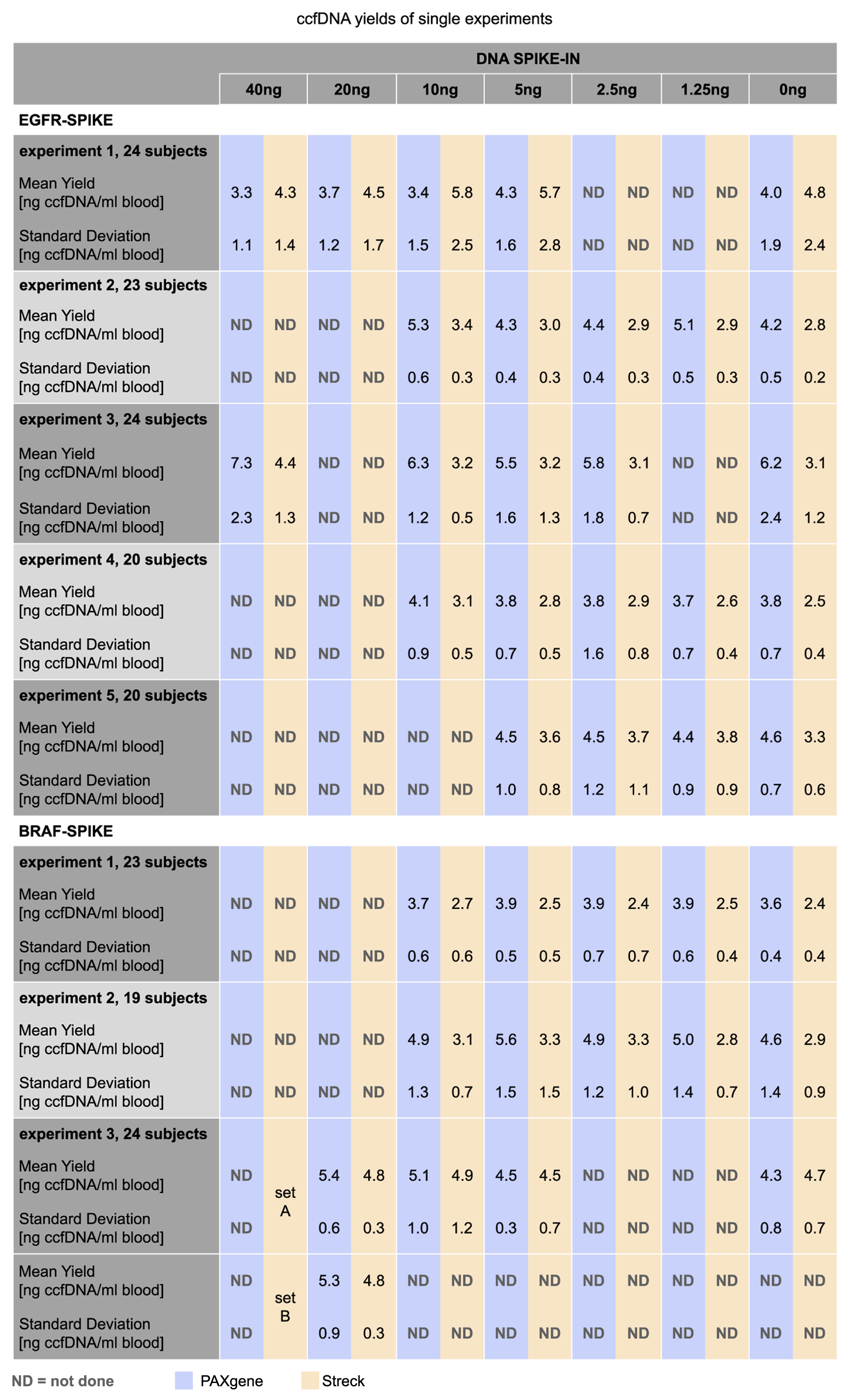

Supplement: S1 Table — Yields were determined by using a Qubit 2.0 Fluorometer and Qubit™ 1x dsDNA HS Assay-Kit. (TIF) [file pone.0253401.s002.tif]
